# Acceptability and Satisfaction Associated With the Introduction of the PrePex Circumcision Device in Maputo, Mozambique

**DOI:** 10.1097/QAI.0000000000000764

**Published:** 2016-05-24

**Authors:** Beverley Cummings, Edgar Necochea, Thais Ferreira, Benilde Soares, Mehebub Mahomed, Humberto Muquingue, Leonel Nhambi, Debora Bossemeyer, Tigistu A. Ashengo

**Affiliations:** * Division of Global HIV/AIDS, Centers for Disease Control and Prevention (CDC), Maputo, Mozambique;; † Jhpiego, Baltimore, MD; and; ‡ Jhpiego, Maputo, Mozambique.

**Keywords:** male circumcision, devices, HIV prevention, Mozambique

## Abstract

**Background:**

Adult device circumcision may potentially reach more men in Sub-Saharan Africa, with fewer human resource and capacity needs than surgical procedures. Despite these advantages, little is known about device acceptability, including pain and maintaining the device in situ.

**Methods:**

Healthy, HIV-negative men, between 18 and 49 years, in a Maputo clinic, were consecutively asked to participate in a circumcision device study that included assessing acceptability. Clinical forms and self-administered surveys were used to collect data at various times during the circumcision process for consenting men. Data were entered into a central database and analyzed using statistical software.

**Results:**

Between May and July, 2013, 504 men received device circumcision. Placement was painless for 98.2% of the male population, but the pain was more common during removal with 38.3% reporting severe or unbearable and 21.5% moderate pain. Satisfaction was high at both time points with 88.8% and 92.6% of men being very or somewhat satisfied at placement and removal, respectively. Half of the male population (50.2%) was very or somewhat comfortable with the device in situ; whereas, 36.8% were somewhat or very uncomfortable. Common device difficulties experienced were painful erections (38.5%) and difficult urination (21.8%) and hygiene (21.4%). By the final clinic visit at day 49, 90.4% of them were very or somewhat satisfied with the procedure.

**Discussion:**

High levels of satisfaction were reported for device circumcision, despite the pain noted during removal and some challenges with the device in situ. Given the advantages and acceptability among Mozambican men in this study, device circumcision could be offered, when clinically appropriate, as an alternative to surgery.

## INTRODUCTION

Male circumcision (MC) has been practiced by many communities in Mozambique for centuries, and, in addition to religious significance, circumcision is often served as a rite of passage to adulthood and was performed during adolescent initiation schools.^1,2^ Approximately 51% of the Mozambican male population, aged 15–49 years, are circumcised; however, there is significant variation across the nation's 11 provinces. HIV prevalence is consistently lower in the provinces where MC is commonly practiced and higher where fewer men are circumcised.^3^

MC services for HIV prevention were initiated in Mozambique in November 2009 with the Ministry of Health (MOH) setting a goal of circumcising 2 million male persons between the ages of 10 and 49 years by the end of 2017.^4^ More than 440,000 male persons have been circumcised through December 2014,^5^ however, progress on scaling up the intervention has been slower than expected, and expansion has been hampered by challenges related to human resources and infrastructure. Recent technological advances, such as circumcision devices, provide viable options for addressing some of the operational challenges faced by resource-limited countries like Mozambique, while possibly reaching more of the male population with MC services.^6^

Circumcision devices are widely used for infants with great success, but experience with adolescents and adults has been limited, particularly in Sub-Saharan Africa, where rapid expansion of MC programs for HIV prevention is most urgent. One nonsurgical option for adult MC is PrePex, an elastic collar compression device. Although various studies have determined the safety of devices like PrePex,^7,8^ there is less information about device circumcision acceptability among men, which may vary by a number of factors including sociodemographics.^9^ This article describes a PrePex study that was conducted, in part, to assess the acceptability of the device and procedure in Mozambique. We sought to document attitudes towards and experiences with device circumcision (eg, acceptability during the procedure and during the healing period with the device in situ), effects on daily activities, pain, and satisfaction with final cosmetic results. These data will assist the MOH with recommendations on the use of the device in adult MC programs, including demand creation messages and comprehensive counseling and informational materials.

## METHODS

### Study Design and Procedures

Our prospective cohort study recruited healthy, HIV-negative men, between the ages of 18 and 49, who were accessing standard surgical circumcision services at the Jose Macamo Health Center in Maputo, Mozambique. Men seeking MC were consecutively asked about their interest in PrePex device circumcision and participation in the study after receiving detailed information about this alternative method. Potential participants received a medical history screening and a genital examination conducted by the PrePex providers, to determine that they were in overall good physical health. Potential participants also were required to consent for HIV counseling and testing. Serological testing for HIV was conducted using the Mozambique MOH national algorithm with Determine HIV-1/2 and Uni-Gold HIV rapid test kits. Men with contraindications for device circumcision (eg, genital or penile conditions such as physical abnormalities, active infections, prepuces with limited flexibility bleeding disorders, and diabetes) or who were HIV-positive were not permitted to participate and were referred for standard surgical circumcision, if appropriate.

Enrollment was limited to potential participants who were capable of providing informed consent, understood and agreed to the study procedures and requirements (eg, abstaining from sexual intercourse and masturbation for 6 weeks after removal, returning to the health care facility for follow-up visits, permitting photographs of the circumcision process, and agreeing to complete surveys and interviews), lived within 25 km of the facility, provided valid contact information (ie, a working telephone number, address of residence, and place of employment), were able to communicate in Portuguese, and had a penis that fit into one of the 5 PrePex ring sizes.

Eligible men received PrePex counseling before device placement. PrePex procedures were performed at the Jose Macamo Health Center by 4 nurses. The nurses were trained and certified to perform PrePex circumcisions for this study by PrePex Master Trainers and certified PrePex doctors in Mozambique. The PrePex device was removed on day 7 at the health center and the men returned for clinical follow-up on days 28 and 49. We sought to enroll and circumcise 500 men.

### Study Measures and Instruments

Providers completed detailed case report forms (CRFs) to collect data on all clinical processes, outcomes, and adverse events on days 0 (placement) and 7 (removal), and to assess wound healing during the 2 follow-up clinic visits that were 28 and 49 days after device placement. The study team also conducted follow-up telephone calls on days 2, 14, and 21 after placement to assess pain, discomfort, healing, and adherence to medical instructions, including sexual abstinence. Pain was measured using a visual analog scale (VAS) with 6 levels (0, 2, 4, 6, 8, and 10) from least to most painful and was assessed during placement, while the device was in situ, during removal, and after removal. The clinical data were supplemented by a self-administered survey that all men completed at 5 time points: before placement, immediately after placement, 7 days after placement (after device removal), 28 days after placement, and 49 days after placement. Clinical outcomes are presented elsewhere in this journal edition (see “Safety and Efficacy of the PrePex Male Circumcision Device: Results from Pilot Implementation studies in Mozambique, South Africa and Zambia”).

### Data Management and Analysis

Data on the circumcision procedure collected during clinic visits and telephone calls were recorded by hand on the study CRFs. Once verified by the study's clinical supervisor, data from the CRFs were digitally entered using CSPro software version 3.3 (Washington, DC) and analyzed using SPSS (Armonk, NY). Paper surveys were completed by clients, and these data were entered and processed in SPSS. All data were double entered to ensure accuracy.

### Ethical Considerations

Participants provided written informed consent before participating in study activities. Reimbursement for participation was offered as compensation for the time required to complete surveys and transportation costs to return to the clinic for follow-up visits. The total value of the reimbursement was estimated at approximately US $25. The study protocol was approved by the National Bioethics Committee for Health (*Comité Nacional de Bioética para a Saúde*) of Mozambique, Johns Hopkins University, and the Center for Global Health in the US Centers for Disease Control and Prevention.

## RESULTS

A total of 608 men were evaluated to participate in this study and 104 of them were determined to be ineligible. Reasons for ineligibility included HIV infection (n = 65, 62.5%), other medical conditions (eg, phimosis) (n = 16, 15.4%), and inability to adhere to study procedures (eg, no cell phone) (n = 23, 22.1%). A sample of 504 men received PrePex circumcision between May and July, 2013. Approximately 70% of the men circumcised using the PrePex device were 24 years of age or younger and not married. Most men (58.7%) were employed at the time of circumcision and 35.7% were students. Men selecting device circumcision were well educated with 84.1% attaining a secondary or postsecondary education. The demographic characteristics of the study sample are included in Table 1.

**TABLE 1.**
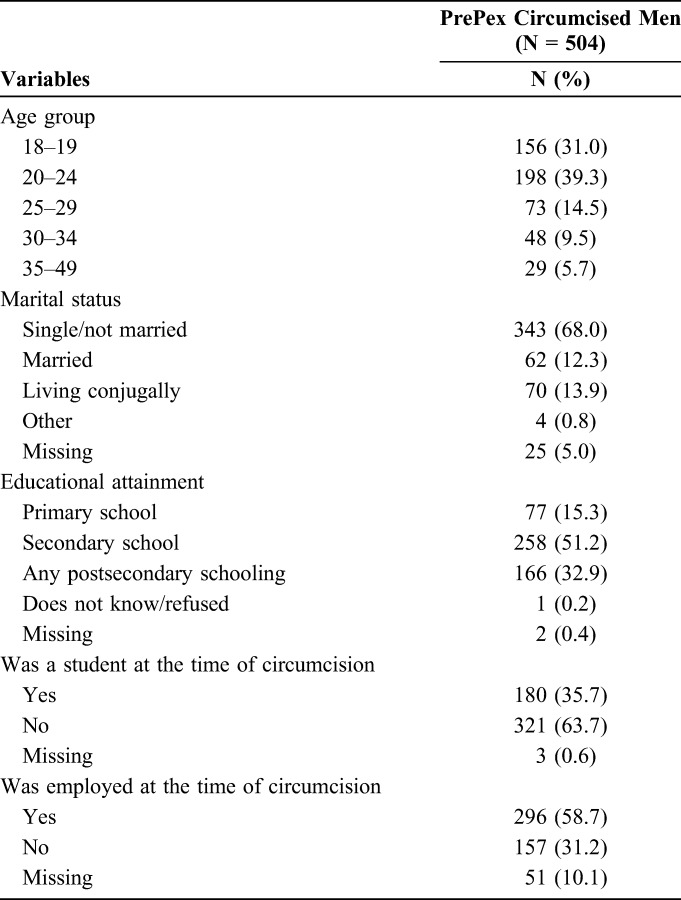
Sociodemographic Characteristics of Men Circumcised With the PrePex Device in Maputo, Mozambique, 2013

Details on pain and overall satisfaction throughout the PrePex circumcision process and required clinical follow-up are presented in Table 2. Pain on day 0 was assessed at 3 sequential time points: during placement (presented in the table) and 15 and 60 minutes after placement. The actual placement was painless for almost all men (98.2%). Perceived pain was minimal for men at 15 (n = 503) and 60 (n = 48) minutes after placement at 99.2% and 97.9%, respectively. Pain was also measured at 3 points on day 7, including before, during (presented in the table) and after removal. Before device removal, 91.0% of men reported no or slight pain (VAS 0 and 2), and 2.6% experienced severe or unbearable pain (VAS 8 and 10). Pain levels increased notably during removal with 38.3% of men reporting severe or unbearable pain (VAS 8 and 10), and 21.5% of men reporting moderate pain (VAS 6); however, the reported pain lessened within minutes. After removal, the level of pain decreased, with 54.1% of men reporting no or slight pain; however, 14% remained with severe or unbearable pain. The level of pain on days 28 and 49 remained very low, with 96.3% and 98.5% of men, respectively, reporting no pain.

**TABLE 2.**
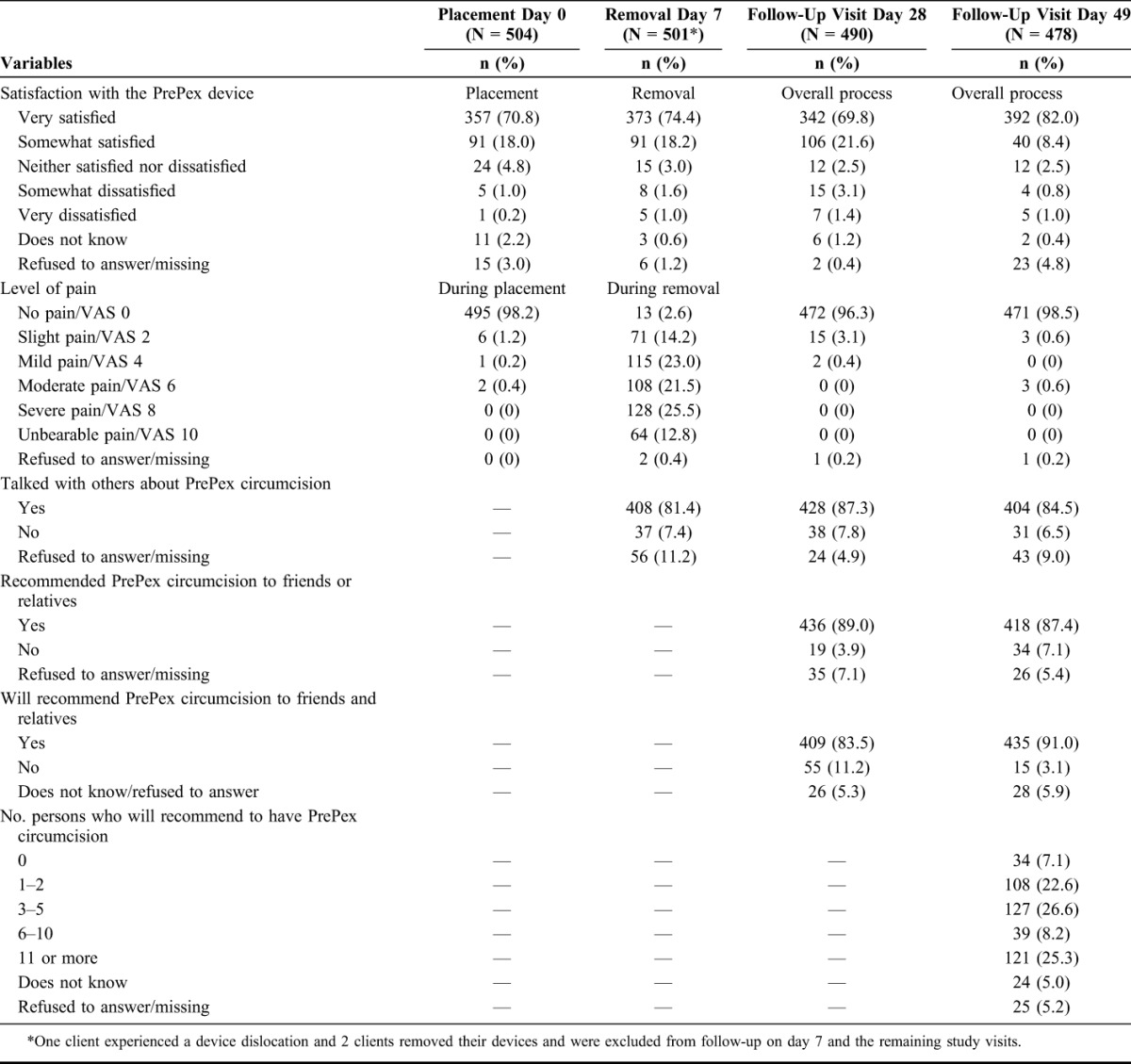
Pain and Satisfaction With PrePex Circumcision by Men in Maputo, Mozambique, 2013

Related to satisfaction, 88.8% of men reported being very or somewhat satisfied with the placement (day 0), and 92.6% reported similar levels of satisfaction with the removal procedure (day 7). Satisfaction remained high on days 28 and 49, with 91.4% and 90.4% of men reporting to be very or somewhat satisfied, respectively. Only 4.5% of men on day 28 and 1.8% on day 49 were somewhat or very dissatisfied with the overall circumcision process. By study completion, more than 80% had talked had about PrePex. Of these men, approximately half (49.5%) talked about PrePex with friends, followed by female partners (36.9%), family members (7.8%), and work colleagues (2.7%). Additionally, by day 49, approximately 90% of men had or intended to recommend PrePex.

Table 3 presents data collected after removal on day 7 that summarizes experiences with the PrePex device in situ. Among all men, half (50.2%) were very or somewhat comfortable wearing the device and similar comfort levels were reported regardless of age. In contrast, 36.8% of all men said that they were very or somewhat uncomfortable with the device in situ, ranging from 34.5% among 20- to 24-year-old to 41.5% of 18- to 19-year old men. Study providers instructed the participating men to gently clean their penis twice per day using warm water and mild soap. Following these instructions was very or somewhat easy for 63.6% of all men, but 22.6% of them said that maintaining their hygiene was a little difficult. Most men found that performing routine activities (eg, dressing) and working or attending school was very or somewhat easy, 59.7% and 67%, respectively. PrePex-related absence from work or school was limited to 1 day or less for 60.4% of the employed men and students. Older men were less likely to take time off because of the procedure (*P* = 0.025). The most common difficulty experienced during the 7 days was painful or uncomfortable erections, which was reported by 38.5% of all men. Additionally, almost equal proportions of men indicated that they had difficulties urinating because of meatal narrowing (21.8%) and cleaning their genitals (21.4%); 29.9% of all men reported that they did not experience any difficulties. There were statistically significant differences by age for urination difficulties (*P* = 0.037) and for men who did not report difficulties (*P* = 0.011). Few men (3.5%) said that they had sexual intercourse or masturbated while wearing the device.

**TABLE 3.**
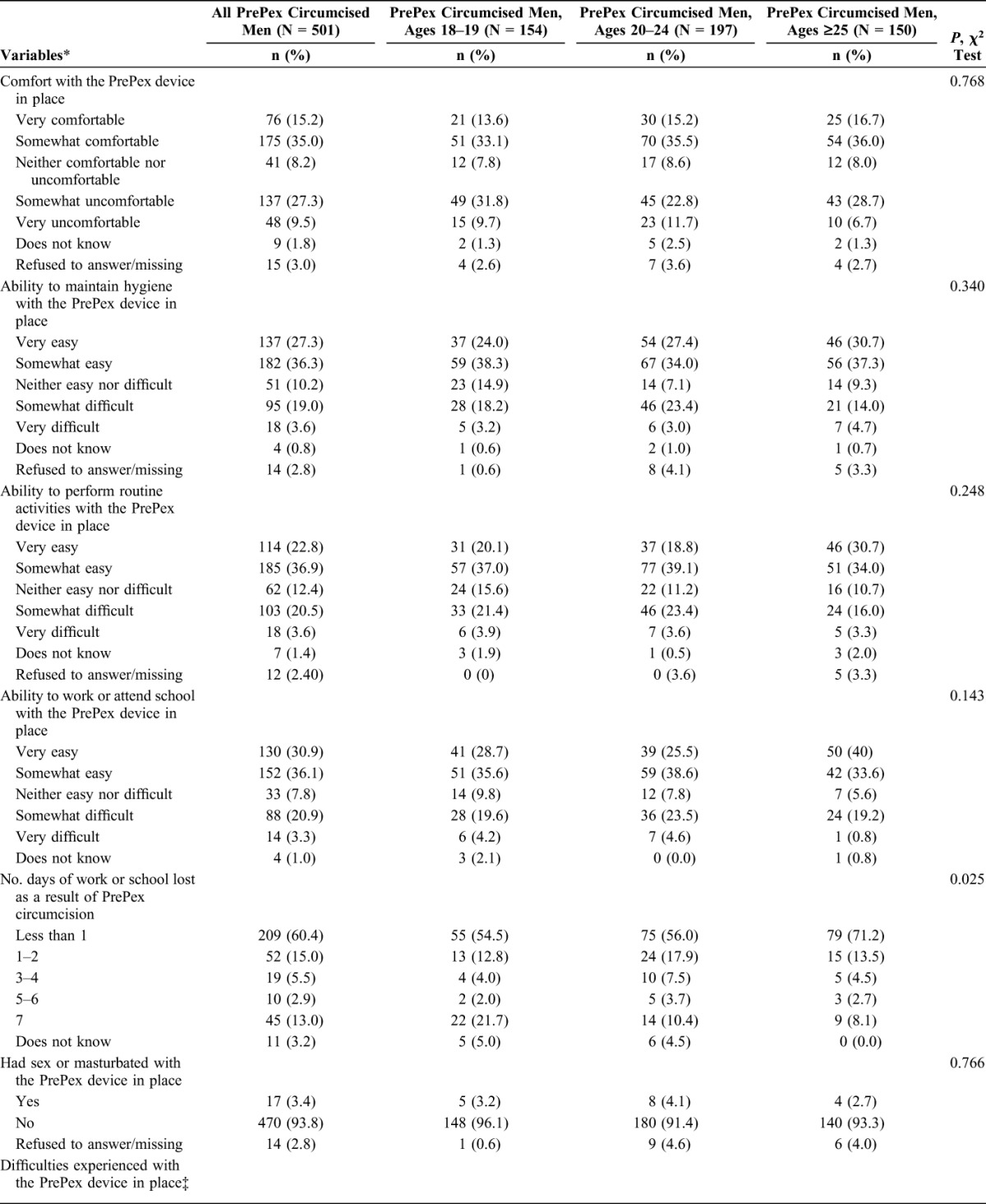

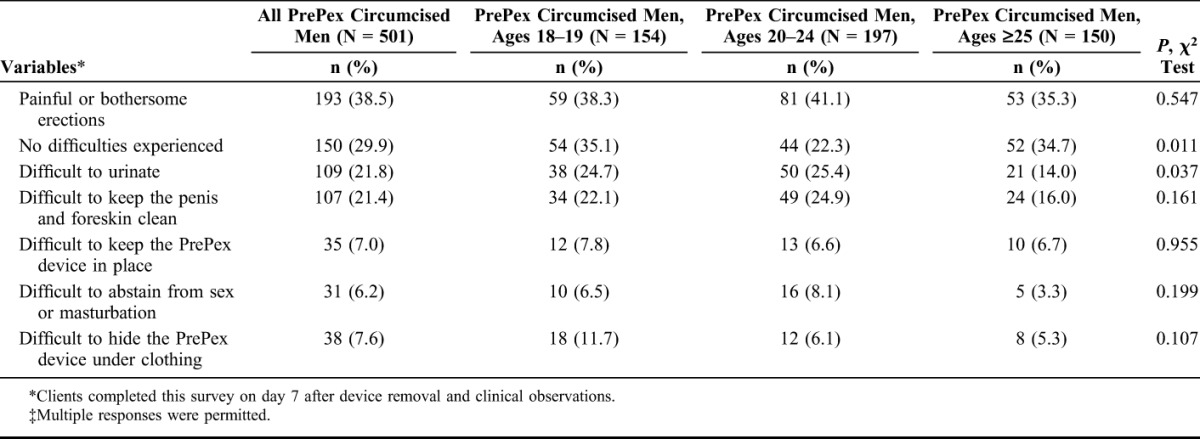
Experiences With the PrePex Circumcision Device In Situ (Days 0–7) Among Men by Age in Maputo, Mozambique, 2013

A multiple logistic regression model measuring the relationship between PrePex satisfaction after complete healing at day 49 found that satisfaction was neither influenced by specific demographic variables (eg, age, student and employment status, educational attainment, or marital status) nor the pain level at device removal.

## DISCUSSION

Our study documented a high level of satisfaction with PrePex device circumcision and the associated processes among Mozambican men in Maputo. At placement, 70.8% of men were very satisfied and 18% were somewhat satisfied; of note, this increased slightly to 74.4% and 18.2% of men, respectively, at removal. Although the proportion that reported being very satisfied had diminished by day 28, by day 49 the proportion increased to a high of 82.0%. We believe that the moderate improvement in the reported satisfaction levels observed on day 7 was related to the relief of device removal; similarly, the level of satisfaction on the last day of follow-up is likely because healing was either complete or nearly complete. We further considered this final satisfaction measure to be an indication of a high degree of contentment with the final circumcision outcome. We explored the acceptability of pain and having the device in situ for 7 days, which we hypothesized would have the most impact on procedure acceptance among the general male population. Published literature from Rwanda and Kenya has shown that men experience some pain at specific points throughout the device circumcision process, most notably at night with erections and when the device is removed.^6,7,10^ This was also evident in our data. More than 90% of men said that they did not experience pain, and there were no clients that had a lot or unbearable pain at placement. During the device removal, the reported pain intensified, but lessened within minutes and was insignificant for almost all men during the 2 clinical follow-up visits at days 28 and 49. Association between satisfaction and pain was neither observed in our logistic regression nor by the key demographic variables. This finding was unexpected. We hypothesized that students and employed men would be highly satisfied with PrePex because of the fact that, in theory, less time off is required and that men who experienced less pain at removal would experience greater satisfaction. Conversely, we initially believed that married men would potentially have less satisfaction because of the slightly longer, necessary abstinence period. We found that maintaining the device in situ for 7 days did not pose a challenge for most men in our study, who reported that they were able to urinate, clean their genitals, and abstain from sex and masturbation. In fact, in older men greater than 25 years of age, who would be the likely priority for demand creation of device circumcision, relatively few difficulties were reported.

More than 8 in 10 device circumcised men talked with others about PrePex. By day 49, 87.4% of men had already recommended PrePex, and 91.0% of them said that they would recommend it in the future. Experiences in Mozambique, where demand creation for voluntary medical male circumcision was initially restricted, have shown that interpersonal communication is one of the most important factors for encouraging men to access services. This has also been validated by other research and documented in other settings,^11,12^ and likely will be critical for the device's successful introduction as a tool for voluntary medical male circumcision scale-up.

Several implementation and programmatic factors posed limitations to this study. The study was conducted at 1 site in a periurban area of the capital, Maputo. As a result, acceptability in more rural areas and other regions of the country remains unknown. Because the MOH considers expansion to ensure that there are no cultural or social barriers to the acceptance of device circumcision, similar rapid field studies in other Mozambican locations will be necessary. Our study population was fairly young with similar demographics, therefore it is difficult to know how 25-year-old and older men will respond to device circumcision. However, the small number of older men circumcised through this study found it to be an acceptable procedure and these men were some of the most satisfied and comfortable people with the device. Another study-related limitation is that we collected very minimal information from men who refused or were ineligible for device circumcision as a basis for comparison. However, it is noteworthy that our study successfully recruited for PrePex predominantly among men seeking surgical circumcision who had no previous information about devices. Other limitations related to study implementation included the lack of validation of the pain scale before the start of the study; lower follow-up rates for telephone calls on days 2 (85.9%), 14 (80%), and 21 (88.2%) in comparison to clinic visit follow-ups, which ranged from 95.4% to 100%; and varying completeness of the self-administered surveys that were answered after long clinic visits. It is difficult to ascertain and quantify the degree to which these issues may have biased the data. Reported pain levels were consistent with those found in other studies.^8,10^ Although lower than desired, the telephone response rate was better than participation rates for epidemiologic studies conducted in the United States, including the Behavioral Risk Factor Surveillance Survey,^13^ and there were no key differences among men noted in incomplete questionnaire responses. Furthermore, during routine program services that include device circumcision, study-related issues (eg, survey completeness and follow-up retention) will not be relevant.

Despite these limitations, we believe that the inclusion of device circumcision as part of the national program would be well accepted by Mozambican men. Similar acceptance of PrePex has been observed in Rwanda, where the device has been the primary method of circumcision for national HIV prevention efforts, and in Kenya, where the high acceptability of PrePex circumcision was reported despite some client concerns about odor and pain during removal.^8,14^ Our study provides enhanced understanding of the satisfaction associated with the device and its procedures, which will assist with developing communication materials to support this circumcision method. Specifically, information is needed that emphasizes practical strategies for maintaining hygiene and fostering realistic expectations about potential difficulties, including pain at removal and with erections. Demand creation should capitalize on the enthusiasm for device circumcision observed among men in our sample and use their willingness to talk about and recommend PrePex to mobilize other men. Additionally, our study underscores the need for exploration of pain management techniques to assist men with the discomfort experienced at various points in the circumcision process. Finally, the ongoing monitoring of factors associated with PrePex acceptability is warranted as active surveillance begins in many countries and we recommend incorporating qualitative methodologies to explore aspects of the process that are acceptable/satisfactory and unacceptable/unsatisfactory.
